# Prevalence, Pattern and Functional Outcome of Post COVID-19 Syndrome in Older Adults

**DOI:** 10.7759/cureus.17189

**Published:** 2021-08-15

**Authors:** Sathyamurthy P., Sudha Madhavan, Viswanathan Pandurangan

**Affiliations:** 1 Internal Medicine, Sri Ramachandra Institute of Higher Education and Research, Chennai, IND

**Keywords:** elderly, postcovid syndrome, covid-19, older adults, anxiety, depression

## Abstract

Introduction

COVID-19 pandemic has been shown to produce high infection rates, significant morbidity and mortality among older adults. A significant proportion of people who have recovered from acute COVID-19 illness seem to suffer from post COVID-19 syndrome. Post COVID-19 syndrome is thought to be a multisystem disease involving physical, functional, mental and psychological domains. This analysis tries to estimate the prevalence, pattern and functional outcomes of post COVID-19 syndrome in hospitalized older adults.

Methods

A prospective cohort study was done on 279 older adults who were discharged from our centre between August 1st, 2020 and November 30th 2020. Information was collected through a telephonic interview after 90 days of discharge from the hospital using a standard questionnaire by a trained physician. Collected data were analyzed with IBM SPSS Statistics for Windows, Version 23.0 (IBM Corp., Armonk, NY).

Results

After 90 days of recovery, the most common symptoms prevalent in the study population were fatigue (8.9%) followed by cough (4.3%), breathlessness (1.8%), dizziness (1.4%), myalgia (1.1%), loss of smell and taste (0.8%) and chest discomfort (0.7%). The prevalence of risk of anxiety in the study population after 90 days of recovery was 7.5% while that of risk of depression was 12.2%. After 90 days of recovery, 66 (23.6%) patients reported the presence of at least one clinical feature while 9.3% had two or more clinical features. On comparing the mean activities of daily living (ADL) 5.58 (.795) and mean instrumental activities of daily living (IADL) 5.84 (1.49) before the illness and 90 days following recovery there was no statistical difference for the study population.

Conclusion

The prevalence of post COVID-19 syndrome in older adults is about 9.3%. The most common symptoms reported by older adults after 90 days following recovery were fatigue followed by cough and breathlessness. Most older adults retained their baseline functional status after 90 days of recovery from acute COVID-19.

## Introduction

The COVID-19 pandemic, which began in December 2019, has produced high infection rates as well as significant morbidity and mortality among older adults [1-3]. Immune senescence and age-related immune remodeling might be the reason for this vulnerability [4]. Six months into the pandemic, reports of persisting symptoms and long-term morbidity in COVID-19 survivors have been published across the world [5-13]. Most of the cases have symptoms that persist from the time of acute clinical illness, whereas some cases have symptoms that appear even after recovery [5,7] from acute COVID-19 and can also be intermittent [7]. Residual organ damage, persistence of systemic inflammation, the effects of hospitalization, and associated comorbidities might be the contributing factors for this [5,14]. Slowly, researchers have started using the term “long COVID-19 syndrome” or “post-COVID-19 syndrome” for these protracted symptoms following virological recovery of COVID-19 [7,8,10,12,14-18]. Post-COVID-19 syndrome is thought to be a multisystem disease [6,7,14,18], involving physical (breathlessness, fatigue, and anosmia), functional (reduced activity), mental (cognitive impairment), and psychological (anxiety and depression) domains [7,8,9,11,13,16,19,20]. Nevertheless, the precise definition or the duration of post-COVID-19 syndrome remains unclear [7,10,14,16,18]. Moreover, the prevalence of various symptoms of post-COVID-19 syndrome, the features of the study population, and the timing of analysis have varied across the study settings [14,18]. In December 2020, guidelines from the National Institute of Health and Care Excellence suggested a temporal criterion of 12 weeks (approximately three months) for defining post-COVID-19 syndrome [15]. Studies have shown that this syndrome affects a significant proportion of recovered COVID-19 patients, independent of their age, clinical severity, and laboratory features [6,12-14,18,19]. However, an earlier comprehensive review suggested that post-COVID-19 syndrome is likely to occur more among women and older adults [7,18]. Data in the older adults are lacking, yet it is essential for providing appropriate post-COVID-19 rehabilitation in this vulnerable age group. This study aimed to analyze a cohort of older adults hospitalized with COVID-19 for the presence, prevalence, and patterns of post-COVID-19 syndrome alongside their functional outcomes 90 days after their recovery and discharge from the hospital.

## Materials and methods

This study included patients at least 65 years old who were hospitalized with acute COVID-19 and were discharged in a stable condition after recovery between August 1, 2020 and November 30, 2020. COVID-19 was confirmed in these patients using nasopharyngeal swab real-time polymerase chain reaction testing. Patients were clinically categorized as either mild to moderate illness (M/M) or severe to critical illness (S/C) [21]. Mild illness was defined as having any of the various signs and symptoms of COVID-19 but without shortness of breath, dyspnea, or abnormal chest imaging. Moderate illness was defined as having evidence of lower respiratory disease during clinical assessment or imaging and with oxygen saturation (SpO2) ≥ 94% on room air at sea level. Severe illness was defined as having SpO2 < 94% on room air at sea level. Critical illness was defined as having respiratory failure, septic shock, and/or multiple organ dysfunction [21].

**Figure 1:**
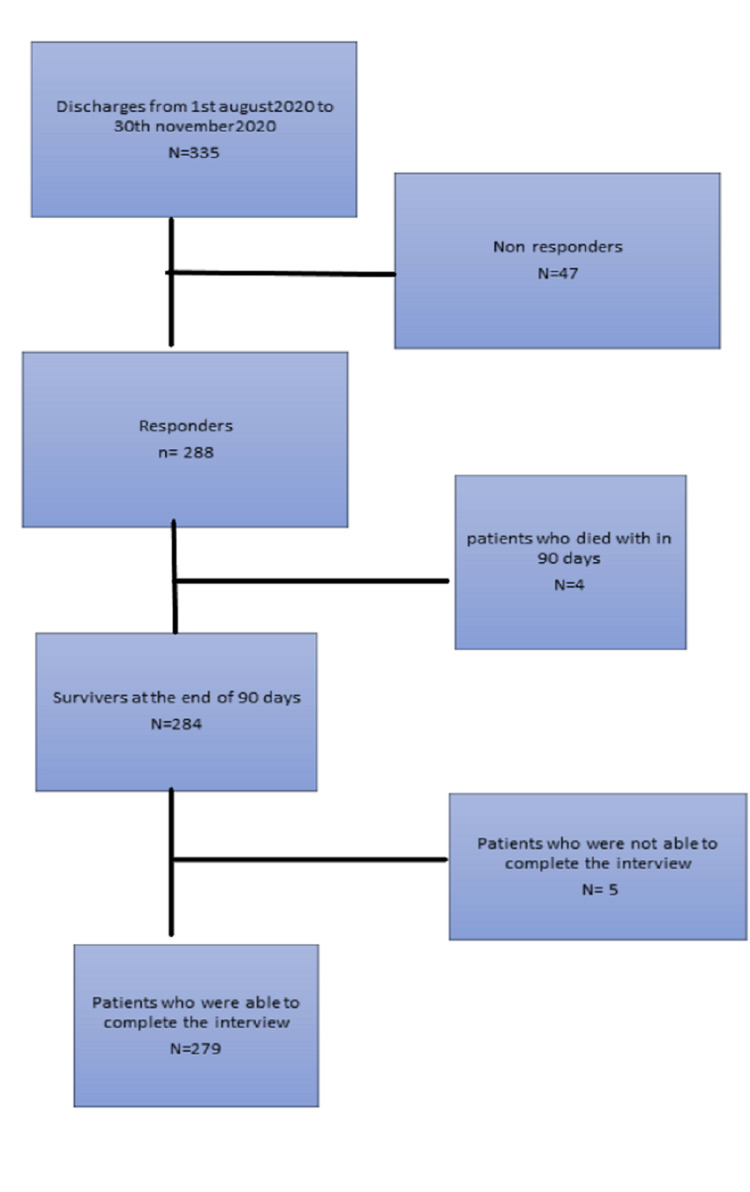
Patient flow chart

A telephone interview was attempted using a standard questionnaire (Appendix 1) by a trained physician 90 days from the date of discharge. Patients who responded within five days (i.e., from day 91 to 96 post-discharge) after a maximum of five attempts and completed the interview were included in the analysis. Non-responders due to various reasons, patients who died before the survey, and patients who developed significant medical illness (such as aphasia) before the survey, which precluded them from completing the interview, were all excluded from the study. Out of 335 cases in the study period, 47 did not respond because of various reasons (not willing to participate, not answering the call, etc.), four died within 90 days after discharge, and five were unable to complete the interview due to medical illness. Finally, 279 eligible older adults were included in our analysis (Figure 1).

The questionnaire (Supplementary Questionnaire 1) was composed of details regarding the following: symptoms persisting and present at the end of 90 days; comorbidities present during hospitalization; KATZ index of independence in activities of daily living (ADL) [22], scores and LAWTONS instrumental activities of daily living (IADL) [23], scored before the illness and 90 days after discharge from the hospital; mental status screening using the Short Portable Mental Status Questionnaire (SPMSQ) [24]; screening for depression using a 5-item version of the Geriatric Depression Scale [25]; and screening for anxiety using a short form of the Geriatric Anxiety Inventory [26].

Because the concept and components of post-COVID-19 syndrome were in the evolving stage at the start of this study, SPMSQ was conducted only for patients who presented with altered mentation upon hospital admission.

The collected data were analyzed with IBM SPSS Statistics for Windows, Version 23.0 (IBM Corp., Armonk, NY). In the descriptive statistics frequency analysis, categorical variables were described as percentages, whereas continuous variables were described as mean ± standard deviation (SD). Unpaired sample t-test was used to find significant differences between the bivariate samples among independent groups. Chi-square test was used to find significance in categorical data. Similarly, if the expected cell frequency is <5 in 2 × 2 tables, then Fisher's exact test was used. Statistical significance was set at p < .05.

As per the government regulations, this study has been registered with the Clinical Trial Registry of India (CTRI) (ref no. CTRI/2020/11/029264). The study was approved by the Sri Ramachandra Institute of Higher Education and Research (SRIHER) institutional ethics committee, ref. no IEC-NI/20/SEP/75/70(COVID19).

## Results

Out of 279 patients analyzed in the study, there were 178 (63.8%) men and 101 (36.2%) women (Table 1), with no significant differences in mean ages between the two (71.1 vs. 71.3, respectively). Clinically, 163 (58.4%) patients were considered M/M, whereas 115 (41.6%) were S/C (Table 1). The difference in the prevalence of severity categories between male and female groups was not statistically significant (p = 0.449). The most common symptoms in the study population were fever (74.6%), followed by cough (35.8%), breathlessness (24%), fatigue (21.8%), and myalgia (19.7%) (Table 1). There were no significant differences in the prevalence of symptoms between men and women. Among the comorbidities, hypertension (HTN, 58.4%) was the most prevalent, followed by diabetes mellitus (DM, 52.7), coronary artery disease (CAD, 20.8%), and hypothyroidism (7.5%) (Table 1). Hypothyroidism was significantly more prevalent among women than in men (14.9% vs. 3.4%, respectively; p = 0.0005).

**Table 1:** Baseline characters of the study population. M/M: mild to moderate clinical illness; S/C: severe to critical clinical illness

Variable	Male n (%)	Female n (%)	P-value	Total n (%)
Numbers	178 (100)	101 (100)		279 (100)
Mean age in years (SD)	71.1 (5.43)	71.3 (5.54)	.682	71 (5.56)
Diabetes mellitus	104 (58.4)	43 (42.6)	.11	147 (52.7)
Hypertension	96 (53.9)	67 (66.3)	.043	163 (58.4)
Coronary artery disease	39 (21.9)	19 (18.8)	.540	58 (20.8)
Cerebrovascular disease	3 (1.7)	4 (4)	.259	7 (2.5)
Dementia	1 (0.6)	1 (0.6)	1.000	2 (0.7)
Chronic kidney disease	8 (4.5)	1 (1)	.163	9 (3.2)
Hypothyroidism	6 (3.4)	15 (14.9)	.0005	21 (7.5)
Obstructive airway disease	7 (3.9)	4 (4)	1	11 (3.9)
Clinical severity of Covid-19				
M/M	101 (56.7)	62 (61.4)	.449	163 (58.4)
S/C	77 (43.3)	39 (38.6)	.449	116 (41.6)
Symptoms reported during admission				
Fever	134 (75.3)	74 (73.3)	.711	208 (74.6)
Headache	8 (4.5)	4 (4)	.405	12 (4.3)
Cough	68 (38.2)	32 (31.7)	.275	100 (35.8)
Breathlessness	46 (25.8)	21 (20.8)	.222	67 (24)
Fatigue	40 (22.5)	21 (21.8)	.277	61 (21.8)
Anorexia	19 (10.7)	7 (6.9)	.301	26 (9.3)
Loss of smell and taste	14 (7.9)	9 (8.9)	.718	23 (8.3)
Vomiting	5 (2.8)	4 (4)	.727	9 (3.2)
Diarrhoea	15 (8.4)	7 (6.9)	.656	22 (7.9)
Chest discomfort	3 (0.6)	1 (1)	.521	4 (1.4)
Odynophagia	5 (2.8)	3 (3)	1.000	8 (2.9)
Myalgia	33 (18.5)	22 (21.8)	.783	55 (19.7)
Dizziness	4 (2.2)	1 (1)	.509	5 (1.8)
Altered mentation	3 (1.7)	1 (1)	.639	4 (1.4)

**Figure 2:**
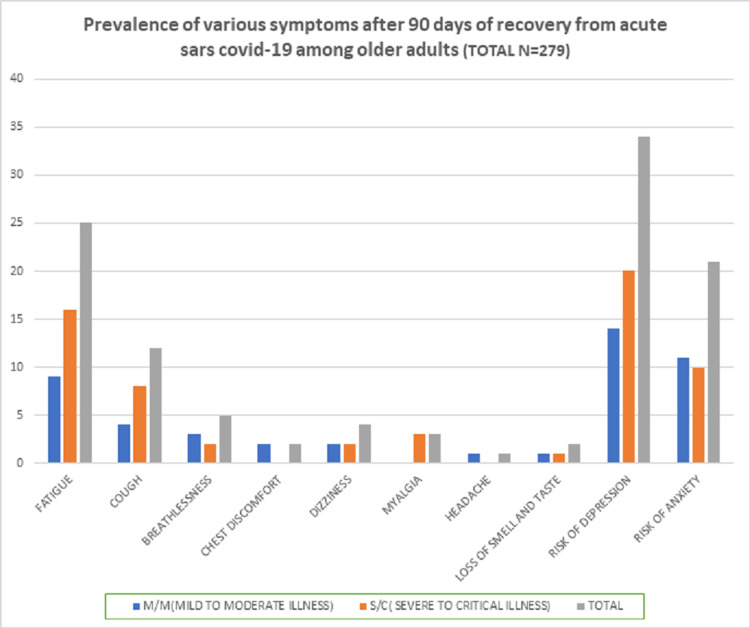
Prevalence of various symptoms 90 days post recovery from acute COVID-19 among older adults (n = 279).

The most common symptom 90 days after discharge was fatigue (8.9%) (Table 2). The other persisting symptoms were cough (4.3%), breathlessness (1.8%), dizziness (1.4%), myalgia (1.1%), loss of smell and taste (0.8%), and chest discomfort (0.7%) (Table 2 and Figure 2). There was no difference in the prevalence of persisting symptoms between men and women.

**Table 2:** Prevalence of various symptoms 90 days post recovery from acute COVID-19 illness among older adults. M/M: mild to moderate clinical illness, S/C: severe to critical clinical illness

Variable	Male n (%)	Female n (%)	P-value	M/M n (%)	S/C n (%)	P-value	Total n (%)
Number	101 (100)	178 (100)		163 (100)	116 (100)		279 (100)
Mean age in years (SD)	71.1 (5.43)	71.3 (5.54)		70.6 (4.96)	72 (6.03)		71 (5.46)
Headache	0 (0)	1 (1)	.405	1 (0.6)	0 (0)	.586	1 (0.4)
Cough	10 (5.6)	2 (2)	.275	4 (2.5)	8 (6.9)	.0005	12 (4.3)
Breathlessness	4 (2.2)	1 (1)	.222	3 (1.8)	2 (1.7)	.0005	5 (1.8)
Fatigue	16 (9)	9 (8.9)	.277	9 (5.5)	16 (13.8)	.077	25 (8.9)
Myalgia	2 (1.1)	1 (1)	0.783	0 (0)	3 (2.6)	.116	3 (1.1)
Loss of smell and taste	2 (1.2)	0 (0)	.718	1 (0.6)	1 (0.9)	.518	2 (0.8)
Chest discomfort	1 (.6)	1 (1)	.521	2 (1.2)	0 (0)	.120	2 (0.7)
Dizziness	4 (2.2)	0 (0)	.509	2 (1.2)	2 (1.7)	.036	4 (1.4)
Risk of depression	23 (12.9)	11 (10.9)	.618	14 (8.6)	20 (17.2)	.0219	34 (12.2)
Risk of anxiety	17 (9.6)	4 (4)	.102	11 (6.7)	10 (8.6)	.559	21 (7.5)

In comparing the prevalence of persisting symptoms between clinical severity groups, cough was significantly more prevalent in the S/C group versus the M/M group (6.9% vs. 2.5%, respectively) (Table 2). Ironically, the prevalence of persistent breathlessness was higher in the M/M group than in the S/C group (1.8% vs. 1.7%, respectively) (Table 2 and Figure 1). There was no difference in the prevalence of the other symptoms between the groups.

On the basis of the screening questionnaire, 90 days after recovery, the prevalence of anxiety risk was 7.5%, whereas that of depression risk was 12.2% (Table 2). There was no statistical difference in the prevalence of anxiety and depression risks between men and women. However, the prevalence of depression risk was significantly more (17.2% vs. 8.6%) in the S/C group than in the M/M group 90 days after recovery (p = 0.0219) (Table 2). There was no significant difference in the prevalence of risk of anxiety between the two groups (6.7% vs. 8.6%) (Table 2).

Regarding the presence of clinical features, 90 days after recovery, 66 (23.6%) patients reported the presence of at least one clinical feature. Among these 66 patients, only 26 (9.3%) had two or more clinical features (Table 3). The prevalence of post-COVID-19 syndrome (two or more clinical features) was significantly high among S/C patients (14% vs. 6%) when compared with M/M patients (p = 0.50) (Table 3).

**Table 3:** Prevalence of post Covid syndrome among older adults. M/M: mild to moderate illness; S/C: severe to critical illness

Variable	M/M, N = 163 (100%)	S/C, N = 116 (100%)	P-value	Total N = 279 (100%)
One clinical feature	21 (12.9%)	19 (16.4%)	.050	40 (14.3%)
Two or more clinical features	10 (6.1%)	16 (13.8%)	.050	26 (9.3%)
Total	31 (19%)	35 (30.2%)		66 (23.6%)

The baseline mean ADL and mean IADL of the study population 90 days after recovery were 5.57 ± 0.279 and 5.77 ± 1.556, respectively (Table 4). There was no significant difference between mean ADL 5.58 (±.795) and IADL 5.84 (±1.49) before the illness (Table 4).

**Table 4:** Comparison of mean ADL and IADL before illness and after 90 days of recovery. ADL: Activities of daily living; IADL: Instrumental activities of daily living.

Variable	Before illness	90 days after post recovery	P-value
Mean ADL (STD)	5.58 (.795)	5.57 (.279)	.805
Mean IADL (STD)	5.84 (1.49)	5.77 (1.556)	.325

## Discussion

Our analysis included 279 patients, with 178 (63.8%) men. Most of the earlier studies on hospitalized older adults with acute COVID-19 have reported males outnumbering the females in the frequency of admissions [1,8,27,28]. The most common presenting symptoms in the study population were fever (74.6%), cough (35.8%), breathlessness (24%), fatigue (21.8%), and myalgia (19.7%). Earlier reports have all shown a similar spectrum and prevalence of symptoms among hospitalized older adults with COVID-19 [1,27,28]. As seen with the earlier studies on COVID-19 affecting older adults [1,8,27,28], the most prevalent comorbidities in our study population were HTN (58.4%), DM (52.8%), and CAD (20.8%) (Table 1).

The most common persisting symptom reported 90 days after recovery was fatigue (8.9%). The other symptoms reported were cough (4.3%), breathlessness (1.8%), dizziness (1.4%), myalgia (1.1%), loss of smell and taste (0.8%), and chest discomfort (0.7%) (Table 2 and Figure 2). This spectrum of post-COVID-19 symptomatology is similar to that of previous reports [7,14,18].

Despite these similarities, many earlier studies conducted on post-COVID-19 hospitalized patients have reported a very high frequency of symptoms, with >50% of patients reporting at least one symptom [8,9,11,13,19]. The lesser prevalence of symptoms in our study could be because most of the earlier studies analyzed a heterogeneous population with a smaller sample size. An application-based COVID-19 symptom study showed that the frequency of post-COVID-19 symptoms declines over time, with the prevalence of symptomatic patients decreasing from 13% to 2.3% at four to 12 weeks post-recovery, respectively [7]. Hence, the decreased prevalence of symptoms in our study is understandable, because we analyzed patients 90 days after recovery, in contrast to earlier studies that had follow-up periods of <60 days [8,9,11,13,19].

Anxiety and depression have been recognized as a part of post-COVID-19 syndrome by many authors [8,14,20,29], but these have been reported with varying rates [8]. A recent review has estimated the prevalence of anxiety and depression to be between 23% and 26% among post-COVID patients [14], and another study reported the prevalence of anxiety and depression to be 29.6% and 26.8%, respectively [20]. Our study showed far less prevalence of anxiety and depression (7.5% and 12.2%, respectively) in older adults after recovery from acute COVID-19 (Table 2 and Figure 2). This could be due to our larger sample size and longer follow-up period, especially because most of the earlier studies did their analysis within eight weeks of recovery.

When comparing the clinical features between the clinical severity groups, S/C patients were more likely to have persistent cough and breathlessness along with a higher risk of depression (P = 0.0219) 90 days after recovery from acute illness compared with M/M patients (Table 2 and Figure 1).

The prevalence of post-COVID-19 syndrome has widely varied between reports. In a recent review [18], the prevalence rate varied widely between 4.7% and 80%, with the temporal criteria for analysis varying between two and 24 weeks. In another report [14], the prevalence was as high as 80% in hospitalized patients, compared with non-hospitalized patients at 10%, with the overall prevalence estimated to be 10%. In a Mediterranean cohort study [12], the incidence of post-COVID-19 syndrome (defined as the presence of at least one symptom) was 58.2% in cases with severe pneumonia, compared with 37% in cases without pneumonia.

Our study defined post-COVID-19 syndrome as the presence of at least two clinical features >12 weeks post-recovery [15]; its overall presence was approximately 9.3% among older adults in our study (Table 3). This lower prevalence could be due to the better sample size and longer follow-up period because the prevalence of post-COVID-19 syndrome decreases over time [7]. Comparing patients by clinical severity, post-COVID-19 syndrome was significantly higher in the S/C group than in the M/M group. This is in line with an earlier cohort study [13] that showed that patients with severe pneumonia had a higher incidence of post-COVID syndrome.

The functional status of patients after recovery from acute COVID-19 is expected to be poor because of various reasons like fatigue, myalgia, and breathlessness [5,7-9,19]. So far, the functional status of older adults after recovery from COVID-19 has not been objectively assessed. In our analysis of 279 patients, there was no significant difference in mean ADL and IADL before the illness and at 90 days after recovery (Table 4). Therefore, it can be said that older adults regained their near-baseline function status 90 days after recovery from acute COVID-19.

Strengths and limitations

To the best of our knowledge, this is the first study of post-COVID-19 syndrome exclusively conducted on older adults with appropriate temporal criteria. Only 14% (47) of patients were non-responders during the follow-up survey. Moreover, unlike earlier studies, we included psychiatric sequelae and functional status in our analysis.

However, our study also has limitations. First, this was only a single-center study conducted during the first wave of COVID-19 in India. Mental status screening was performed during follow-up only for patients who presented with altered mentation during admission. Last, some parts of the questionnaire used for the interview are subject to recall bias.

## Conclusions

The prevalence of post-COVID-19 syndrome in the elderly is approximately 9.3%. The most common symptom reported 90 days after recovery was fatigue, followed by cough and breathlessness. A significant proportion of the elderly had a risk of anxiety and depression 90 days after recovery and discharge from the hospital. Older adults seem to regain their baseline functional status in terms of ADL and IADL 90 days after recovery from COVID-19. However, because this was a single-center study conducted during the first wave of COVID-19, more comprehensive and multi-centric studies are required to accurately estimate the prevalence of post-COVID-19 syndrome. Because approximately 23.6% of patients had at least one symptom persisting 90 days after recovery, it is important to establish appropriate follow-up protocols such as setting up post-COVID-19 clinics to address the needs of this vulnerable population.

## Appendices

Supplementary Questionnaire 1

Telephonic interview and assessment

Patients id-

Age -

Sex-

Date of discharge-

Date of interview-

Interviewer-

Clinical severity- Mild/Moderate/Severe/Critical

Section A - Physical (√   the relevant category)

Symptoms since discharge                    

1.     Dyspnoea                         

Persisting/Newly developed persisting/Newly developed resolved /Resolved/NA             

2.     Cough                                                       

Persisting/Newly developed persisting/Newly developed resolved /Resolved/NA             

3.     Chest pain/discomfort                                

Persisting/Newly developed persisting/Newly developed resolved /Resolved/NA             

4.     Odynophagia

Persisting/Newly developed persisting/Newly developed resolved /Resolved/NA             

5.     Fever                                                                          

Persisting/Newly developed persisting/Newly developed resolved /Resolved/NA             

NA             

6.     Myalgia                                                                    

Persisting/Newly developed persisting/Newly developed resolved /Resolved/NA             

7.     Headache                                                                  

Persisting/Newly developed persisting/Newly developed resolved /Resolved/NA             

8.     Diarrhoea                                                 

Persisting/Newly developed persisting/Newly developed resolved /Resolved/NA             

9.     Vomiting                                                                 

Persisting/Newly developed persisting/Newly developed resolved /Resolved/NA             

10.  Anorexia                                                  

Persisting/Newly developed persisting/Newly developed resolved /Resolved/NA             

11.  Fatigue                                                      

Persisting/Newly developed persisting/Newly developed resolved /Resolved/NA             

12.  Altered mentation                                   

Persisting/Newly developed persisting/Newly developed resolved /Resolved/NA             

13.  Loss of smell and taste                             

Persisting/Newly developed persisting/Newly developed resolved /Resolved/NA             

14.  Dizziness                                                 

Persisting/Newly developed persisting/Newly developed resolved /Resolved/NA             

15.  Any other symptoms (not listed above)

…………                                                                  

Persisting/Newly developed persisting/Newly developed resolved /Resolved/NA             

NA             

Persisting - present during admission and is present till date

Newly developed persisting - NOT present during admission but developed after discharge and still persisting

Newly developed resolved - NOT present during admission, but developed after discharge and resolved now.

Resolved - Present during admission and now resolved

NA-never had these symptoms from hospitalization till date

 Section B-Functional status

ACTIVITIES OF DAILY LIVING (ADL) SCORE: calculated before the illness and at present.

Bathing Points: __________

(1 point) Bathes self completely or needs help in bathing only a single part of the body such as the back, genital area or disabled extremity.

(0 points) Need help with bathing more than one part of the body, getting in or out of the tub or shower. Requires total bathing

Dressing Points: __________

(1 point) Get clothes from closets and drawers and puts on clothes and outer garments complete with fasteners. May have help tying shoes.

(0 points) Needs help with dressing self or needs to be completely dressed.

Toileting Points: __________

(1 point) Goes to toilet, gets on and off, arranges clothes, cleans genital area without help.

 (0 points) Needs help transferring to the toilet, cleaning self or uses bedpan or commode.

Transferring Points: __________

 (1 point) Moves in and out of bed or chair unassisted. Mechanical transfer aids are acceptable

 (0 points) Needs help in moving from bed to chair or requires a complete transfer.

Continence Points: __________

(1 point) Exercises complete self-control over urination and defecation.

 (0 points) Is partially or totally incontinent of bowel or bladder

 Feeding Points: __________

(1 point) Gets food from plate into mouth without help. Preparation of food may be done by another person.

(0 points) Needs partial or total help with feeding or requires parenteral feeding.

Total points: ________ scoring: 6 = High (patient independent) 0 = Low (patient very dependent                                    

Total score

Before illness ……….

At present …………...                                          

INSTRUMENTAL ACTIVITIES OF DAILY LIVING (IADL) SCORE:  calculated before the illness and at present.

Ability to Use Telephone

1. Operates telephone on own initiative;

looks up and dials numbers .......................................................1

2. Dials a few well-known numbers ................................1

3. Answer’s telephone, but does not dial .........................1

4. Does not use telephone at all......................................0

Shopping

1. Takes care of all shopping needs independently ........1

2. Shops independently for small purchases ..................0

3. Needs to be accompanied on any shopping trip ........0

4. Completely unable to shop .........................................0

Food Preparation

1. Plans, prepares, and serves adequate meals independently ...................................................1

2. Prepares adequate meals if supplied with ingredients ..........................................................0

3. Heats and serves prepared meals or prepares meals but does not maintain adequate diet ..........................0

4. Needs to have meals prepared and served ..................0

Housekeeping

1. Maintains house alone with occasion assistance (heavy work) ................................................................1

2. Performs light daily tasks such as dishwashing, bed making ..................................................................1

3. Performs light daily tasks, but cannot maintain acceptable level of cleanliness ....................................1

4. Needs help with all home maintenance tasks ............1

5. Does not participate in any housekeeping tasks ........0

Laundry

1. Does personal laundry completely .............................1

2. Launders small items, rinses socks, stockings, etc........1

3. All laundry must be done by others ...........................0

Mode of Transportation

1. Travels independently on public transportation or drives own car .........................................................1

2. Arranges own travel via taxi, but does not otherwise use public transportation ..........................1

3. Travels on public transportation when assisted or accompanied by another ........................................1

 4. Travel limited to taxi or automobile with assistance of another...................................................0

5. Does not travel at all ...................................................0

Responsibility for Own Medications

1. Is responsible for taking medication in correct dosages at correct time ...............................................1

2. Takes responsibility if medication is prepared in advance in separate dosages ...................................0

3. Is not capable of dispensing own medication ............0

Ability to Handle Finances

1. Manages financial matters independently (budgets, writes checks, pays rent and bills, goes to bank); collects and keeps track of income .............................1

2. Manages day-to-day purchases, but needs help with banking, major purchases, etc ...........................1

3. Incapable of handling money .....................................0
 

Scoring: For each category, circle the item description that most closely resembles the client’s highest functional level (either 0 or 1)

Total score

Before illness ……….

At present ……………

Section C- Comorbidities

Comorbidities Present During Hospitalization (√ the relevant item)

Diabetes mellitus…...

Hypertension …...

Obstructive airway disease…...

Coronary artery disease…...

Cerebrovascular disease…...

Chronic liver disease…...

Chronic kidney disease…...

Connective tissue disease…...

(Rheumatoid arthritis, SLE, MCTD, etc)

Malignancy …...

Recurrent Falls…...

Urinary and faecal incontinence…...

Vision and hearing problems…...

 Comorbidities Developed After Discharge till Date

(√ the relevant item)

Diabetes mellitus…..

Hypertension …...

Obstructive airway disease…...

Coronary artery disease…...

Cerebrovascular disease…...

Chronic liver disease…...

Chronic kidney disease…...

Connective tissue disease…...

(Rheumatoid arthritis, SLE, MCTD, etc)

Malignancy …...

Recurrent Falls…...

Urinary and faecal incontinence…...

Vision and hearing problems…...

Other new comorbidities identified post discharge (not included in the above list)

……………………………………………….

Section D-Cognitive and Mental Status

The Short Portable Mental Status Questionnaire (SPMSQ) verified with the attender (only for patients who presented with altered mentation during hospitalization)

QUESTION   

1. What are the date, month, and year?     Correct Response/ Incorrect Response        

2. What is the day of the week?                Correct Response/ Incorrect Response        

3. What is the name of this place?            Correct Response/ Incorrect Response        

4. What is your phone number?                 Correct Response/ Incorrect Response           

5. How old are you?                                   Correct Response/ Incorrect Response        

6. When were you born?                           Correct Response/ Incorrect Response        

7. Who is the current prime minister?       Correct Response/ Incorrect Response        

8. Who was the prime minister before him?   Correct Response/ Incorrect Response        

9. What was your mother’s maiden name?       Correct Response/ Incorrect Response        

10. Can you count backward from 20 by 3’s?    Correct Response/ Incorrect Response        

Score 0-10 ……………

Section E-Screening for Depression

1. Are you basically satisfied with your life?                                                YES/ NO

2.Do you often get bored?                                                                              YES/ NO

3. Do you often feel helpless?                                                                          YES/ NO

4. Do you prefer to stay at home rather than going out / doing new things?    YES/ NO

5. Do you feel pretty worthless the way you are now?                                     YES/ NO

 Bold answers Score 0-5 ………...

  Section F-Screening for Anxiety

1."I worry a lot of time".                                                       YES/NO

2."Little things bother me a lot”.                                           YES/NO

 3."I think of myself as a worrier”.                                        YES/NO

4."I often feel nervous”.                                                         YES/NO

5."My own thoughts often make me nervous”.                      YES/NO

 Bold answers Score 0-5 ……….
